# Baseline mRNA expression differs widely between common laboratory strains of zebrafish

**DOI:** 10.1038/s41598-018-23129-4

**Published:** 2018-03-19

**Authors:** Lindsay A. Holden, Kim H. Brown

**Affiliations:** Portland State University, Department of Biology, 1719 SW 10th Avenue, Portland, OR 97201 USA

## Abstract

Common strains of wildtype zebrafish (*Danio rerio*) have unique genomic features including SNPs and CNV, but strain information often goes unreported in the literature. As a result, the confounding effects of interstrain variation makes repetition of studies in zebrafish challenging. Here we analyze hepatic mRNA expression patterns between three common zebrafish strains (AB, Tuebingen (TU), and WIK) using Agilent 4 × 44 K gene expression microarrays to establish baseline mRNA expression across strains and between sexes. We observed wide variation in sex-specific gene expression within AB and WIK strains (141 genes in AB and 67 genes in WIK), but no significant variation between sexes within TU. After partitioning the dataset into male and female subsets, we detected 421 unique mRNA transcripts with statistically significant differential expression; 269 mRNA transcripts varied between males, 212 mRNA transcripts varied between females, and 59 mRNA transcripts varied across the three strains, regardless of sex. It is not surprising that mRNA expression profiles differ between sexes and strains, but it is imperative to characterize the differences. These results highlight the complexity of variation within zebrafish and underscore the value of this model system as a valid representation of normal variation present in other species, including humans.

## Introduction

Laboratory strains of zebrafish (*Danio rerio*) have discrete genomic backgrounds; they clade out with very high bootstrap support by distinct SNPs^1^ and have unique sets of copy number variant genomic regions^2^. Because of these genomic traits, zebrafish strains may be able to serve as a proxy to incorporate genetic variation into study design, similar to our understanding of the genomic variation in distinct human populations^3^. The human 1000 Genomes Project found that many common genetic variants are shared across populations, but rarer variants are generally only shared by closely related populations^4^. Analogous to distinct human populations, zebrafish strains have unique origin stories and genetic isolation between strains is maintained by strict husbandry practices.

**Figure 1:**
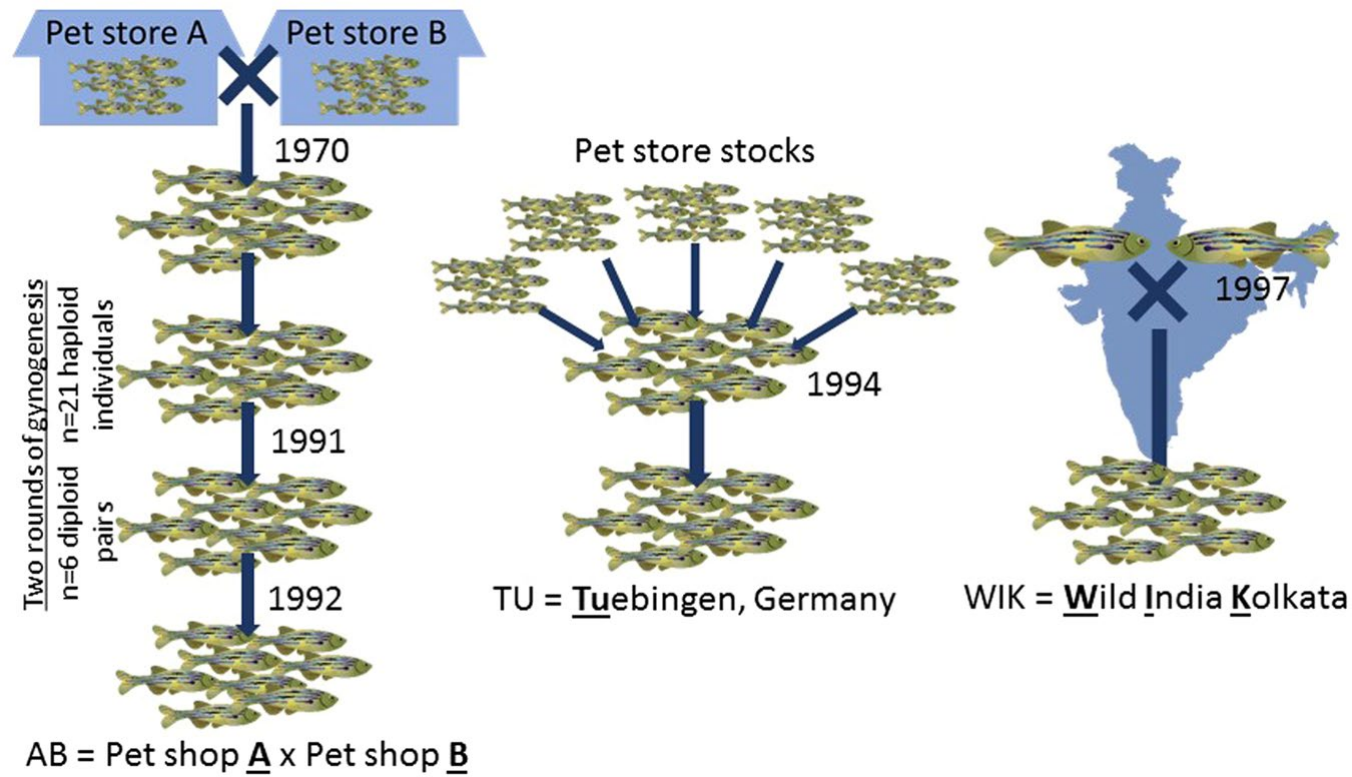
History of strain establishment for common laboratory strains of zebrafish. AB, TU, and WIK are three popular zebrafish strains used in genetic, developmental, and toxicological research with very different origin stories.

Commonly used zebrafish strains such as AB (ZFIN ID: ZDB-GENO-960809-7), Tuebingen (TU; ZFIN ID: ZDB-GENO-990623-3), and WIK (ZFIN ID: ZDB-GENO-010531-2) have well-documented histories (Fig. 1) and are easily obtainable for laboratory manipulations. The AB line began from unknown zebrafish source stocks bought from two pet shops (pet shop A and pet shop B) in Albany, Oregon in the early 1970s^5^. Haploid progeny from AB females were crossed with random AB males for approximately 70 generations until the early 1990s when six diploid progeny stocks (each from a distinct haploid female) were thoroughly intercrossed to produce the modern AB line (sometimes referred to as AB*). The current AB source stock is maintained through large group spawning crosses. The TU strain originated from a composite population of fish purchased from pet shops in 1994 and was maintained as an inbred strain in a lab in Tuebingen, Germany^6,7^. The WIK strain (“Wild India Kolkata”) originated from a single pair mating of wild caught fish in 1997^8^. The establishment and maintenance of these different strains has resulted in a similar observable phenotype.

The high homology between humans and zebrafish—71% of human genes have at least one zebrafish ortholog and 69% of zebrafish genes have at least one human orthologs^9^—makes zebrafish an excellent model to study development, genetics, and toxicology. Unfortunately only 83% of transgenic and 46% of non-transgenic wild type strains of animal models are actually identified in the published literature^10^ indicating that strain-based genetic variation is largely overlooked or ignored. Behavioral traits associated with domestication in wild versus lab-reared zebrafish are associated with differential mRNA expression in the brain^11^, indicating that the genetic isolation and population bottleneck inherent during laboratory strain establishments of zebrafish can create distinct characteristics between strains. Sex is an additional factor that drives differential mRNA expression between strains, mostly associated with hormone biosynthesis^12^. The goal of this study is to identify baseline liver mRNA expression variation between different zebrafish strains and between sexes in support of the growing recognition of normal variation between strains and populations^13,14^ in an organismal and physiological context to support zebrafish as a strong model for translational research.

## Results

### mRNA expression profiles differ between sexes in two of three strains

**Figure 2:**
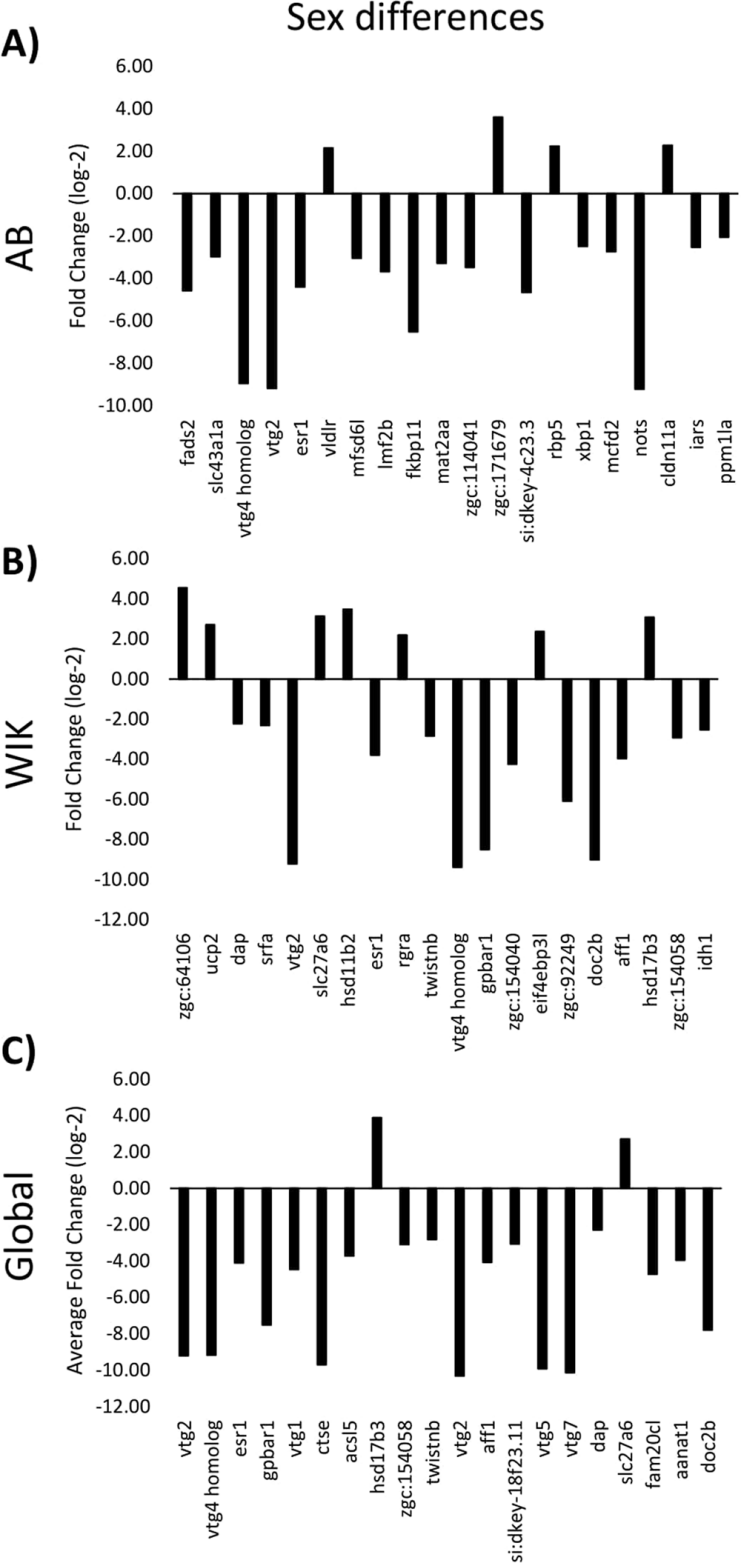
Top 20 most significant differentially expressed genes between sexes. Positive fold change values indicate higher mRNA gene expression in males, as compared to females. Negative fold change values indicate higher mRNA gene expression in females, as compared to males. (**A**) In AB the top 20 q-values range from 0.0049 to 0.0062. (**B**) In WIK the top 20 q-values range from 0.0030 to 0.0150. (**C**) Regardless of strain the top 20 q-values range from 0.0081 to 0.0164. Fold change values were averaged between male and female datasets. See supplementary datasets 1–3 for gene symbol definitions.

Analysis of total hepatic mRNA expression arrays detected 149 probes representing 141 genes that are significantly different between AB males and females (Fig. 2A; Supplementary Dataset 1). Of these, 62 probes have a positive fold change indicating an increased expression of the transcript in males relative to females and 87 have a negative fold change indicating an increased expression of the transcript in females relative to males. Gene ontology analysis of 117 gene IDs (82.98%) mapping to *Danio rerio* shows that differences between males and females in the AB strain occur largely at the endoplasmic reticulum membrane (6.40-fold enriched, q-value = 3.01 × 10^−3^). The biological processes of response to estradiol (87.14-fold enriched, q-value = 2.03 × 10^−2^), cellular response to estrogen stimulus (74.36-fold enriched, q-value = 1.00 × 10^−9^), lipid transport (22.49-fold enriched, q-value = 1.25 × 10^−9^), small molecule biosynthetic processes (10.46-fold enriched, q-value = 8.01 × 10^−4^), and monocarboxylic acid metabolic processes (7.88-fold enriched, q-value = 2.98 × 10^−2^) are statistically over-represented in the dataset and are largely driven by lipid transporter activity (29.05-fold enriched, q-value = 3.99 × 10^−10^) and oxidoreductase activity (4.79-fold enriched, q-value = 9.63 × 10^−4^).

Between WIK males and females, 72 probes representing 67 genes are significantly different (Fig. 2B; Supplementary Dataset 2). Of these, 23 probes have a positive fold change indicating an increased expression of the transcript in males relative to females and 49 have a negative fold change indicating an increased expression of the transcript in females relative to males. Gene ontology analysis of 58 gene IDs (86.57%) mapping to *Danio rerio* shows that differences between males and females in the WIK strain are not restricted to one cellular compartment, but encompass biological processes including response to estradiol (>100-fold enriched, q-value = 2.44 × 10^−3^), cellular response to estrogen stimulus (>100-fold enriched, q-value = 7.38 × 10^−8^), hormone biosynthetic processes (>100-fold enriched, q-value = 1.30 × 10^−2^), and lipid transport (37.80-fold enriched, q-value = 5.23 × 10^−10^). Similar to AB, these over-represented biological processes in WIK are largely driven by lipid transporter activity (42.61-fold enriched, q-value = 3.51 × 10^−8^). Interestingly, at our cutoff values of a minimum of 2-fold change in expression and q-value = 0.05, there are no probes that are significantly different between TU males and females. This is most likely due to a wider variation in the TU gene expression dataset.

Overlapping the differentially expressed probe sets from both AB and WIK produces a set of 40 probes mapping to 36 genes that are differentially expressed between males and females, regardless of strain (Fig. 2C; Supplementary Dataset 3). Of these, only 6 probes have a positive fold change indicating an increased expression of the transcript in males relative to females and 34 have a negative fold change indicating an increased expression of the transcript in females relative to males. Examples of mRNA transcripts conserved across strains include the protein responsible for converting androstenedione to testosterone (*hsd17b3*) in males and an egg yolk precursor (*vtg1-7*) and estrogen receptor (*esr1*), two well-known female-specific transcripts.

### mRNA expression profiles differ between strains

**Figure 3:**
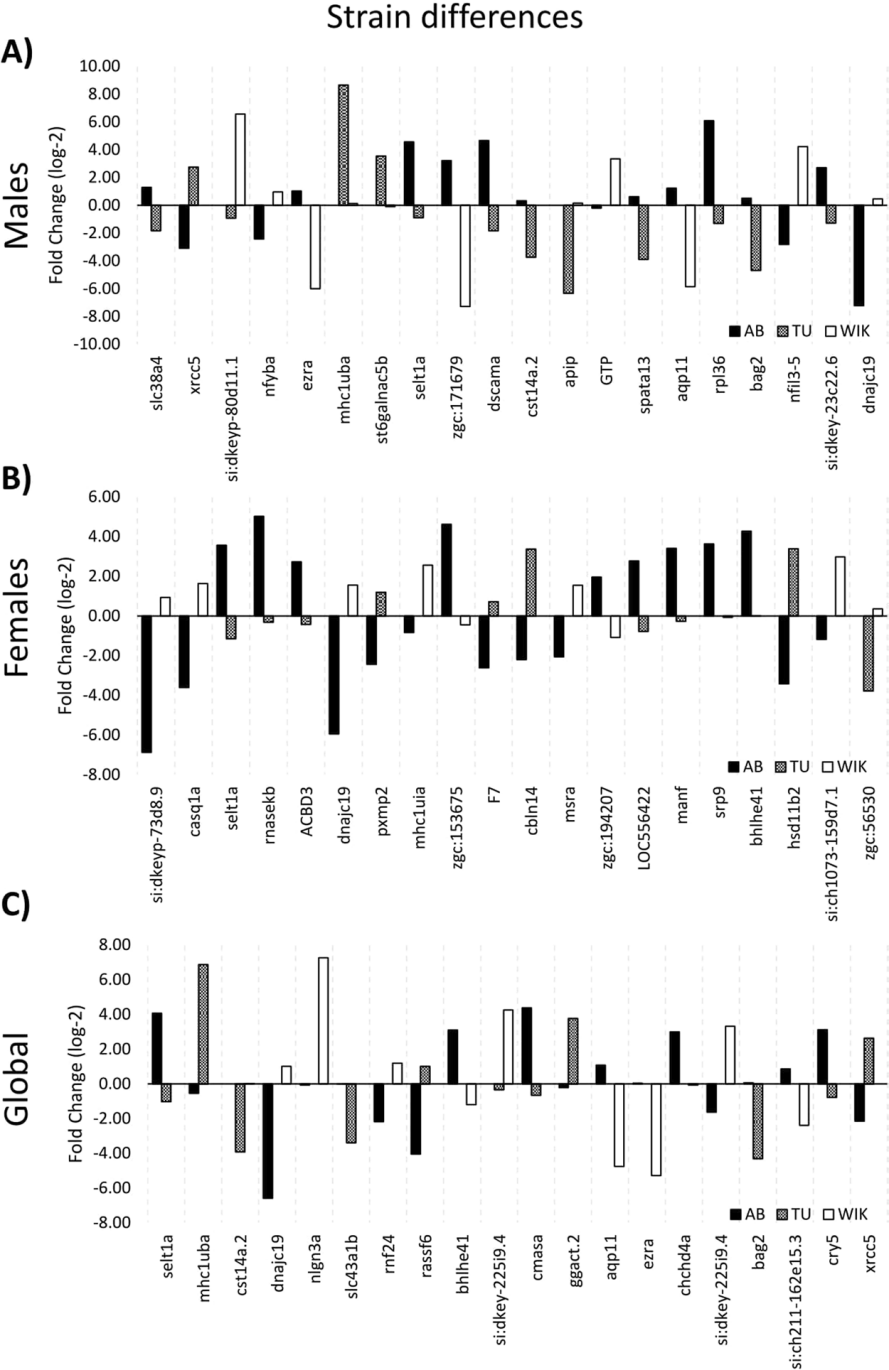
Top 20 most significant differentially expressed genes between strains. Positive fold change values indicate an increase in mRNA gene expression and negative fold change values indicate a decrease in mRNA gene expression. AB is represented by black bars, TU is represented by checkered bars, and WIK is represented by white bars. (**A**) In males the top 20 q-values range from 0.0003 to 0.0020. (**B**) In females the top 20 q-values range from 0.0005 to 0.0014. (**C**) Regardless of sex the top 20 q-values range from 0.0007 to 0.0077. See supplementary datasets 4–6 for gene symbol definitions.

**Figure 4:**
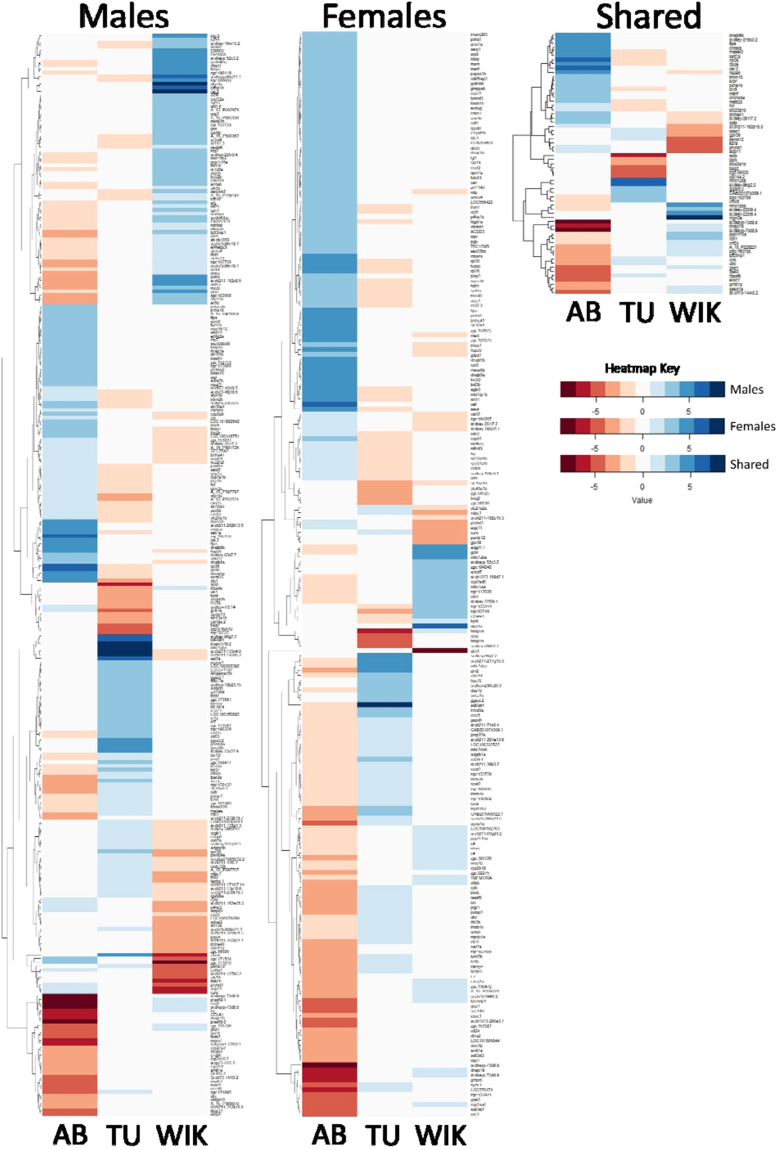
Differentially expressed mRNA transcript heatmaps. Individual heatmaps for males alone, females alone, and shared between the sexes (global) across AB, TU, and WIK strains. Blue indicates a positive fold change in expression, red indicates a negative fold change in expression. Higher saturation indicates stronger positive or negative fold change.

Within males, 292 probes representing 269 genes are significantly different between AB, TU, and/or WIK males (Figs 3A and 4; Supplementary Dataset 4). Seventy-three (73) probes varied between TU and WIK (AB = 0 fold-change), 117 probes varied between AB and WIK (TU = 0 fold-change), and 102 probes varied between AB and TU (WIK = 0 fold-change). Within the strains, the percentage of transcripts with significantly increased expression accounted for 49.2–62.5% of the mRNA transcripts, with a mean of 56.3%. Gene ontology analysis of 237 gene IDs (88.10%) mapping to *Danio rerio* shows that differences between AB, TU, and/or WIK males are not restricted to one cellular compartment or molecular function, but are over-represented by the biological process of circadian regulation of gene expression (45.89-fold enriched, q-value = 7.20 × 10^−3^).

In females, 220 probes representing 212 genes are significantly different between AB, TU, or WIK (Figs 3B and 4; Supplementary Dataset 5). Fifteen (15) probes varied between TU and WIK (AB = 0 fold-change), 80 probes varied between AB and WIK (TU = 0 fold-change), and 125 probes varied between AB and TU (WIK = 0 fold-change). Within the strains, the percentage of transcripts with significantly increased expression accounted for 57.0–67.5% of the mRNA transcripts, with a mean of 60.5%. Gene ontology analysis of 183 gene IDs (86.32%) mapping to Danio rerio shows that differences between AB, TU, and/or WIK females occur largely at the endoplasmic reticulum membrane (5.32-fold enriched, q-value = 9.58 × 10^−4^). Biological processes affected include protein targeting to the endoplasmic reticulum (49.52-fold enriched, q-value = 5.06 × 10^−3^), membrane assembly (31.28-fold enriched, q-value = 3.07 × 10^−2^), and single-organism metabolic processes (2.50-fold enriched, q-value = 3.27 × 10^−3^). These over-represented biological processes are largely driven by catalytic activity (1.72-fold enriched, q-value = 4.34 × 10^−3^).

Overlapping the differentially expressed probe sets from both males and females produces a set of 63 probes representing 59 genes that are differentially expressed between AB, TU, and WIK regardless of sex (Figs 3C and 4; Supplementary Dataset 6). Six (6) probes varied between TU and WIK (AB = 0 fold-change), 29 probes varied between AB and WIK (TU = 0 fold-change), and 28 probes varied between AB and TU (WIK = 0 fold-change). More than 50% of the probes varying between strains, regardless of sex, are attributable to the AB strain alone. Within the strains, the percentage of transcripts with significantly increased expression accounted for 46.7–56.6% of the mRNA transcripts, with a mean of 52.3%. Gene ontology analysis of 52 gene IDs (88.14%) mapping to *Danio rerio* shows no over-representation of any category between AB, TU, and/or WIK, regardless of sex.

## Discussion

**Figure 5:**
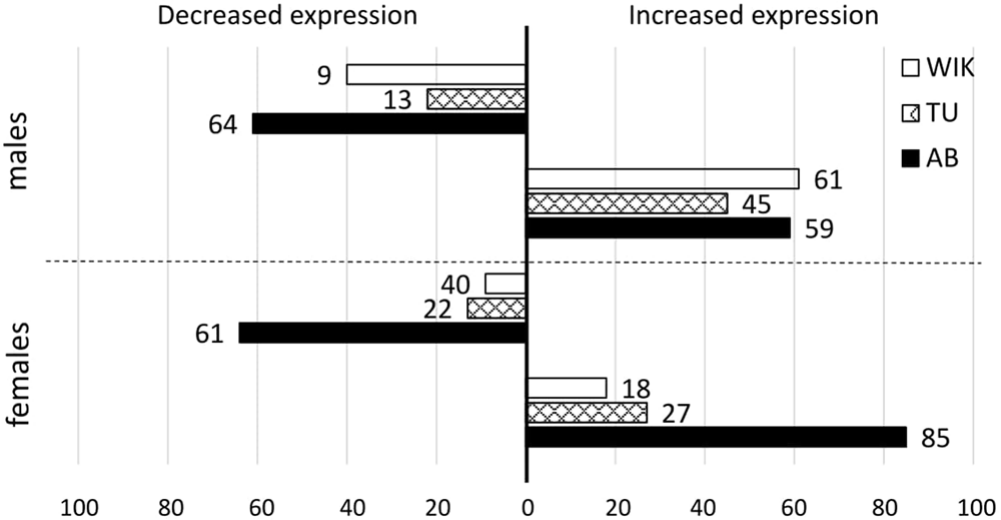
Summary chart of highly differentially expressed probe count in males or females across strains. Each bar represents a count of the mRNA transcripts with 2-fold increased (to the right) or decreased (to the left) expression by strain in males (top) and females (bottom) in AB, TU, or WIK. AB is represented by black bars, TU is represented by checkered bars, and WIK is represented by white bars.

A primary goal of this study was to identify baseline liver mRNA expression variation between different zebrafish strains. We identified large differences between strains, with a majority of differentially expressed mRNA transcripts belonging to AB (Fig. 5). We hypothesize that this is due to the additional bottleneck of gynogenesis in the early establishment of the AB strain and a resulting decrease in heterozygosity by 34%, as similarly observed in gynogenetic diploid rainbow trout (*Oncorhynchus mykiss*)^15^. Additionally, across all sexes and strains, approximately 59% of probes show an increase in expression versus a decrease. The bottleneck of domestication reduces genetic variation^16^, but since there is little to no selection acting on these laboratory strains, we predicted wide variation in expression phenotypes across strains^17^ due to the inherent increase in the inbreeding coefficient^18^. Although we have described robust gene expression variation between AB, TU, and WIK, laboratory stocks still have less diversity between strains when compared to wild-caught zebrafish^19^.

### Sex and strain both drive mRNA expression profiles in zebrafish

**Figure 6:**
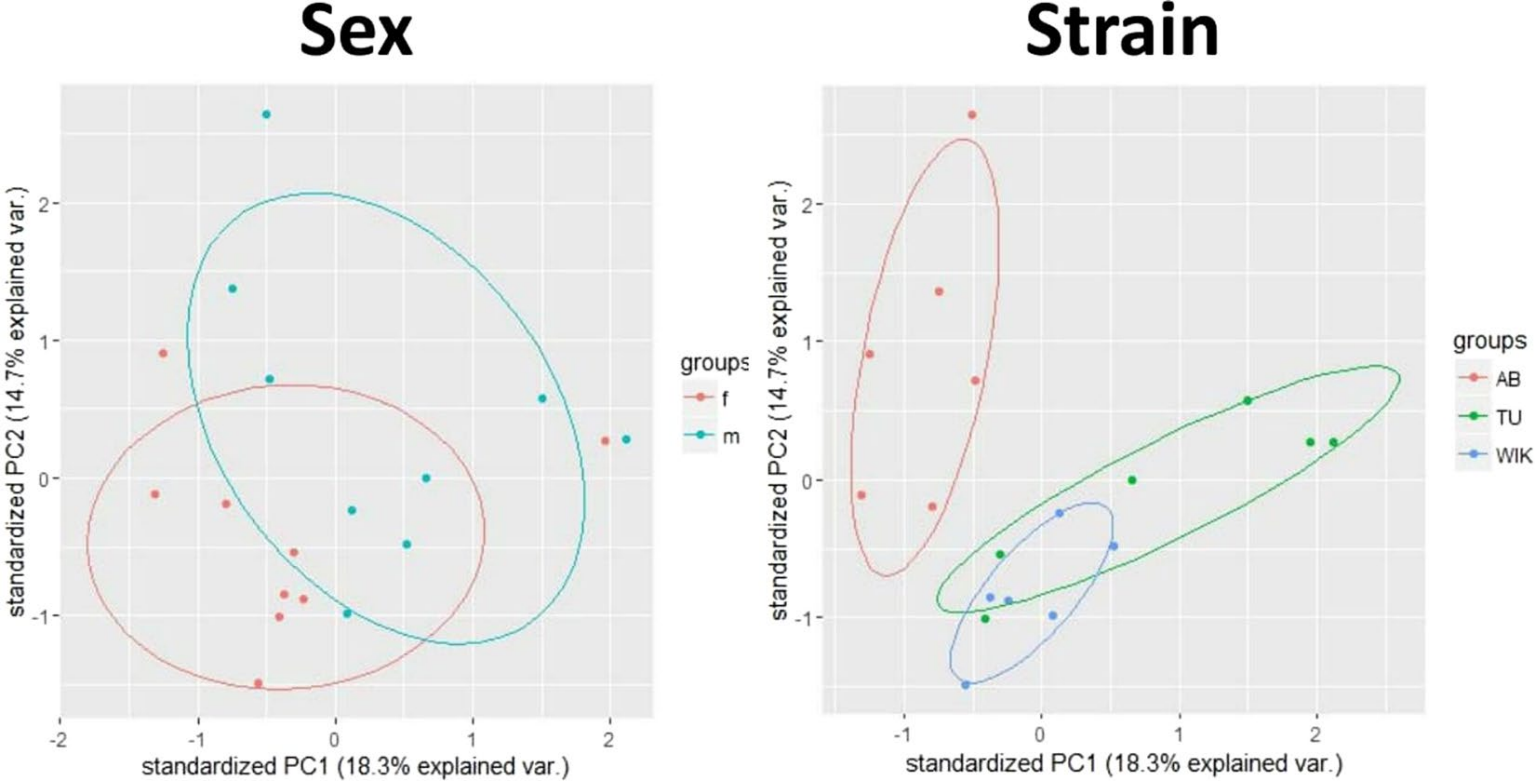
Principle component analysis of samples by sex or strain. PC1 and PC2 explain 33% of the total variance in the dataset. Sample identification by sex shows that male and female samples segregate, with the exception of a single female sample. Sample identification by strain shows that the AB strain clearly segregates from the TU and WIK strains.

Sex determination in zebrafish has been argued extensively in the last decade, but only recently has a six-strain analysis led to a consensus hypothesis. Our current understanding is that genetic factors on chromosome 4 drive the ZW/ZZ sex-determining mechanism, but ultimate sex determination is sensitive to multiple environmental conditions^20^. Fascinatingly, AB and TU strains appear to have lost sex-specific signal across the sex-associated region in chromosome 4, so factors defining male or female development in these strains are still unknown. WIK retains the chromosome 4 sex-associated region and has additional regions on chromosome 14 and several unassembled genomic scaffolds that are associated with sex determination. Interestingly, principle component analysis uncovers male and female grouping, as well as a clear separation of AB away from TU and WIK (Fig. 6). Although sex is a major factor in this dataset, the loss of sex-determining regions in AB and TU do not appear to be driving the difference in mRNA expression between strains. Interstrain variation is most likely due to genetic differences caused by population isolation and bottleneck events during strain establishment. Moreover, we observed a large portion of differentially expressed mRNA transcripts that were specific to the AB strain, probably due to the extreme population bottlenecks and multiple rounds of gynogenesis.

### Lipid transport mRNA transcripts differ between sexes in multiple strains

Genes involved in lipid transport are significantly enriched in differentially expressed mRNA transcripts between males and females in both AB and WIK. Among these are members of the vitellogenin (*vtg1-7*), retinol binding protein (*rbp2a* and *rbp5*), and solute carrier (*slc27a6* and *slc25a48*) families, as well as a transmembrane trafficking protein (*tmed1a*), a kainite glutamate receptor (*grik1a*), and an estrogen receptor (*esr1*). It is important to accurately characterize these differences between strains because lipid transport is critical in chemical messaging, energy storage, temperature maintenance, and formation of membranes, cholesterol and prostaglandins. Furthermore, vitellogenin is a common marker for endocrine disruption in teleosts; any variation between expression in this endpoint may drastically affect interpretation of pharmacological endpoints including estrogenic activity of xenobiotics.

### Circadian rhythm affects mRNA expression in males more than females

Circadian regulation in the zebrafish is directed by light- or dark-induced gene expression in the pineal gland^21^. Although our study design did not control for time of day (AB livers were collected in the morning, while TU and WIK livers were collected in the afternoon), there are only a small number of genes with circadian rhythm annotations in this dataset. Specifically, we identified 295 annotations to 67 genes by searching AmiGO2^22^ annotations for any term including the word “circadian” within zebrafish annotations. In the male dataset there are 6 genes that annotate to circadian rhythm: *arntl1a*, *bhlhe40*, *cry5*, *nfil3-5*, *nfil3-6*, and *nr1d2a*. In the female dataset there are only two genes that annotate to circadian rhythm: *arntl1a* and *cry5*.

To expand our analysis of potential circadian effects on our dataset, we queried circadian rhythm genes annotated in all organisms within AmiGO2. This expanded our list of potential circadian rhythm genes to 2194, but led to no additional genes for the male dataset, and added only one gene, *F7*, to the list of genes present in female dataset that are known to be influenced by circadian rhythm. *F7* has been observed to be regulated by circadian rhythm in the Norway rat^23^ and C57BL/6J mouse^24^, but a similar regulation has yet to be identified in zebrafish. Using a comparable approach, we queried a circadian rhythm RNA-seq dataset in mouse that assessed gene expression in multiple tissues across time^25^. We found 9 genes—*bhlhe41*, *ptgr1*, *dnaja4*, *fads2*, *fkbp5*, *lmbr1l*, *nedd41*, *slc38a4*, and *stk35*—in the mouse circadian rhythm dataset, but as-of-yet there is no clear evidence of oscillation in the expression of these genes in the liver of zebrafish. Again in this vein of inquiry, we queried the circadian expression profiles data base (circaDB)^26^ against 4 mouse liver microarray studies and found 82 genes that overlap our dataset and have evidence of circadian regulation (Supplementary Dataset 7).

Expression patterns for the two circadian genes that are shared between males and females are conserved, with a decrease in expression of *arntl1a* and an increase in expression of *cry5* in the AB strain as compared to TU and WIK. This can be explained by the timing of liver harvest (AB in AM; TU and WIK in PM). What is fascinating, though, are the other genes affected by circadian rhythms that differed in the WIK strain only. *Bhlhe40* had lower expression in WIK and *nfil3-5*, *nfil3-6*, and *nr1d2a* had higher expression in WIK. If expression of these genes were solely driven by circadian rhythms, then we would expect to see similar patterns between TU and WIK. Because this relationship is lacking, we hypothesize that there are other genetic factors that regulate the expression of these genes that differ between strains. This is interesting because experimental design accounts for the differences in males, but females seem to be less sensitive, suggesting that males are more sensitive to circadian perturbation than females. This is not unfounded as sex-specific phenotypes related to circadian rhythm have been observed in several animals, including behavioral traits in Drosophila^27^ and liver metabolism in mice^28^. Most circadian oscillations in gene expression are not conserved across tissues and there are transcriptional “rush hours” prior to dawn and dusk^25^. Our samples were collected starting at 4 hours after dawn and ended 3 hours prior to dusk, which avoids the transcriptional rush hour and minimizes the maximal effects of circadian-driven transcription. Nonetheless, this is a reminder that time of day is a factor that should be considered in zebrafish study design, but that it is not the dominant driver of overall gene expression.

### Functional implications of gene expression variation

While this is solely a descriptive study on the standing variation that exists in three strains of zebrafish, there are functional consequences of variable mRNA expression that should be assessed for the continued application of zebrafish as a model system. For example, in this dataset AB males have a greater than expected number of serine-type endopeptidase (GO:0004252) mRNA transcripts: prss59.1, prss59.2, ela2l, try, and cela1. All of these genes have greater than 7-fold lower expression in AB as compared to TU or WIK. Loss of expression of these genes in AB males may indicate a reduction in their ability to break internal amino acid bonds within polypeptide chains. As another example, WIK males have greater than 7-fold higher expression of two presynaptic membrane assembly (GO:0097105) mRNA transcripts: nlgn3a and cel.2. Both of these genes are involved in neuron cell-cell adhesion and neurexin family protein binding. Neuroligin genes, such as *nlgn3a*, are important in zebrafish nervous system development^29^. Disruption of the neurexin pathway at synapses leads to autistic-like behavior in mice^30^ and mutation in *nlgn3a* in humans was associated with x-linked Asperger and Autism disorders^31^. Moreover, a zebrafish model for autism spectrum disorder displays behavioral differences between strains^32^. *cel.2* is associated with maturity-onset diabetes of the young, type 8, with exocrine disfunction^33^. Because WIK males exhibit higher expression of these genes, they may be compensating for loss of expression of related genes. A functional follow-up would be to see if neuronal synapses are enriched in WIK males for neurexin receptors or if there are any behavioral or exocrine disruption as compared to AB or TU males.

As a final example, AB have an 8-fold decrease in si:dkeyp-73d8.9 mRNA expression, an unknown transcript, in both males and females. Protein-protein alignment of the predicted amino acid sequence for si:dkeyp-73d8.9 against NCBI’s non-redundant protein sequence database indicates that this is most likely a cystatin-like protein. Cystatins are inhibitors of cysteine proteinases and play a role in tumorigenesis, kidney function, and modulation of the immune system^34^. If all AB fish lack expression of this gene, then AB may be a better strain to target for development of mutation strains for model diseases involved in the disruption of the cystatin pathway. Continuation of describing and validating variation within zebrafish is paramount to the expansion of the zebrafish model system. This will further elevate the relevance of zebrafish studies to human health through the incorporation of multiple strains to simulate wide population variances, such as seen in human populations.

## Conclusions

Our current understanding of zebrafish as a genetic model is based on the reference genome, which has only included alternate sequence loci as of June 2017^35^. The addition of alternate loci is a pivotal achievement for zebrafish as a model because it allows the interpretation of datasets with wide variance due to underlying structure within the data, such as genetically distinct sub-groups or populations. This study goes one step further by describing baseline mRNA expression differences between zebrafish strains as a physiological interpretation of established genetic differences between zebrafish strains. We found major differences between strains and sexes including lipid transport and circadian rhythms. In the absence of a practical understanding of intra-population baseline variation, the downstream interpretation of data becomes skewed, reproducibility becomes increasingly challenging, and the application of study results become more abstract. Thus, this study serves as a foundational comparison of the strain-specific variation in mRNA expression in zebrafish and should be used to inform future study designs.

## Methods

### Animal care and husbandry

All zebrafish husbandry and experimental procedures were performed following protocols approved by Portland State University’s Institution Animal Care and Use Committee in accordance with the National Institutes of Health Guidelines for Care and Use of Laboratory Animals and the Public Health Service Policy on Humane Care and Use of Laboratory Animals. Zebrafish are housed on an Aquaneering semi-recirculating housing system at a density of 5 individuals per liter with 10% daily water changes. Water temperature is maintained at 27.5 °C and fish are kept on a 16 hour light, 8 hour dark photoperiod. pH and conductivity are maintained at approximately 7.4 and 1100 µS, respectively. Zebrafish are fed commercial flake food twice daily and supplemented with artemia and rotifer live food. AB, TU, and WIK strains are maintained in-house by random single pair breeding. Larvae are screened for developmental abnormalities and 10 individuals from 25 pairs are randomly selected for the succeeding generation. The fish used in this study were second generation adults originally sourced from ZIRC (Eugene, OR) as batches of 100 embryos. All tissues were collected from healthy adults between 12 and 14 months old. At the time of dissection males weighed 331.7 ± 100.4 mg (mean ± SD) and females weighed 346.6 ± 90.7 mg. Male liver weights ranged from 0.002–0.021% of whole body weight and female liver weights ranged from 0.003–0.028% of whole body weight.

### Nucleic Acid Isolation

White muscle and liver tissues were dissected from 3 males and 3 females from AB, TU, and WIK strains (n = 6/strain; n = 18 total) and disrupted with a mortar and pestle prior to homogenization by passing the samples through a nuclease-free syringe and needle in beta-mercaptoethanol lysis buffer. DNA was extracted on Qiagen DNeasy columns (Qiagen, Valenica, CA, USA) and total RNA was extracted on Qiagen RNeasy columns. Nucleic acid concentrations were determined on a Nanodrop Spectrophotometer 2000 (Thermo Scientific, Wilminton, DE, USA). Both DNA and RNA exhibited high 260/280 ratios of 1.92 ± 0.04 and 2.10 ± 0.03, respectively (average ± SD), indicating adequate quality for downstream analysis.

### mRNA expression arrays

Commercially available 4 × 44 K zebrafish mRNA expression arrays, RNA spike-in kit, and Low Input Quick Amp one-color labeling kit (Agilent) were used following manufacturer’s protocols. In brief, cDNA was synthesized from RNA and transcribed into cRNA using Cyanine-3 fluorescent dCTP. Labeled cRNA was purified using a Qiagen RNeasy mini kit per the manufacturer’s protocol and quantified on a NanoDrop spectrophotometer. Samples with total cRNA yields greater than 1.65 µg and specific activity greater than 6 pmol Cy3/µg were fragmented, hybridized to array slides at 65 °C for 17 hours, washed briefly, and scanned on an Agilent SureScan array scanner using grid file 026437_D_F_20140627 and scan protcol AgilentHD_GX_1Color. Data were extracted from raw TIFF files using FeatureExtraction software (Agilent) and spot brightness values were loaded into R. Raw microarray data files and derived expression values are archived at the Gene Expression Omnibus (https://www.ncbi.nlm.nih.gov/geo) under accession number GSE100583.

### Data normalization, analysis, and annotation

Data were cleaned by subtracting background fluorescence, normalizing across arrays, and averaging duplicate probes within the limma package^36^. Principle component analysis illustrated a clear separation between male and female samples, so all downstream analysis was performed with male and female datasets separated. Within limma, empirical Bayes fitting of a linear model and pairwise contrasts were applied to AB, TU, and WIK strains separately to test for differences between males and females per strain. These results will be referred to as “sex differences”. Similarly, a linear model and pairwise contrasts were applied to males and females separately to test for differences between AB, TU, and WIK per sex. These results will be referred to as “strain differences”. Pairwise comparison values for fold change (log^−2^), average expression (log^−10^), p-value, and q-value were averaged for each strain and centered on zero to facilitate data interpretation. Significant probes were defined as ≥2-fold change in expression and Benjamini-Hochberg^37^ adjusted p-value ≤ 0.05 (q-value). Standard Agilent array annotations were applied to the probes and manually verified across NCBI and Ensembl databases. Conflicting annotations were resolved by direct overlap of mapped probes using UCSC’s LiftOver tool as needed. Heatmaps were produced using the gplots heatmap.2 tool in R. Ordering of genes within heatmaps was performed using Euclidean distances and complete h clustering without scaling.

### Gene ontology analysis

Gene ontology analysis was performed using the Panther Classification Tool^38^ developed and maintained by the Gene Ontology Consortium. Ensembl and NCBI’s ENTREZ gene ID annotations were assessed for statistical over-representation in the *Danio rerio* database (ZFIN last updated 04/2015) using default settings. GO complete annotations (database released 4/24/2017) for cellular component, biological process, and molecular function were assessed with Bonferroni^39^ correction for multiple testing. Genes were considered over-represented at q-value ≤ 0.05 and results are presented as fold enrichment over the *Danio rerio* reference database.

### Ethics approval and consent to participate

All zebrafish husbandry and experimental procedures were performed following protocols approved by Portland State University’s Institution Animal Care and Use Committee in accordance with the National Institutes of Health Guidelines for Care and Use of Laboratory Animals and the Public Health Service Policy on Humane Care and Use of Laboratory Animals.

### Availability of data and material

The datasets generated and analyzed during the current study are available in NCBI’s Gene Expression Omnibus (https://www.ncbi.nlm.nih.gov/geo) under accession number GSE100583.

## Electronic supplementary material

Supplementary dataset descriptions

Dataset 1

Dataset 2

Dataset 3

Dataset 4

Dataset 5

Dataset 6

Dataset 7
