# Supplementary material for: Baseline mRNA expression differs widely between common laboratory strains of zebrafish
Source: Sci Rep. 2018 Mar 19;8:4780. doi: 10.1038/s41598-018-23129-4 (PMC5859126; doi:10.1038/s41598-018-23129-4)
Supplement: Supplementary file 1 — Supplementary dataset descriptions [file 41598_2018_23129_MOESM1_ESM.pdf]

Title

Baseline mRNA expression differs widely between common laboratory strains of zebrafish

Authors

Lindsay A. Holden and Kim H. Brown*

Lindsay A. Holden

Portland State University

Department of Biology

1719 SW 10th Avenue

Portland, OR 97201

E-mail: holdenl@pdx.edu

*Corresponding Author

Kim H. Brown

Portland State University

Department of Biology

1719 SW 10th Avenue

Portland, OR 97201

E-mail: kibr2@pdx.edu

Supplementary Datasets

SupplementaryDataset1.xls

Differentially expressed mRNA transcripts between males and females in AB. File includes probe name, log fold change, average expression, p-value, Bonferroni-corrected p-value, and genomic coordinates (danRer7/Zv9) as reported by Agilent Feature Extract and annotation files. Entrez gene ID, Ensembl ID, gene symbol, and gene name were manually confirmed and harmonized. Gene symbols and names highlighted in pink are differentially expressed in females. Gene symbols and names highlighted in blue are differentially expressed in males.

SupplementaryDataset2.xls

Differentially expressed mRNA transcripts between males and females in WIK. File includes probe name, log fold change, average expression, p-value, Bonferroni-corrected p-value, and genomic coordinates (danRer7/Zv9) as reported by Agilent Feature Extract and annotation files. Entrez gene ID, Ensembl ID, gene symbol, and gene name were manually confirmed and harmonized. Gene symbols and names highlighted in pink are differentially expressed in females. Gene symbols and names highlighted in blue are differentially expressed in males.

SupplementaryDataset3.xls

Differentially expressed mRNA transcripts between sexes regardless of strain. File includes probe name, log fold change, average expression, p-value, Bonferroni-corrected p-value, and genomic coordinates (danRer7/Zv9) as reported by Agilent Feature Extract and annotation files. Entrez gene ID, Ensembl ID, gene symbol, and gene name were manually confirmed and harmonized. Gene symbols and names

highlighted in pink are differentially expressed in females. Gene symbols and names highlighted in blue are differentially expressed in males. Log fold change, average expression, p-values, and Bonferroni-corrected p-values are averaged between AB and WIK differential expression datasets (SupplementaryDataset1 and SupplementaryDataset2).

SupplementaryDataset4.xls

Differentially expressed mRNA transcripts between AB, TU, and WIK in males. File includes probe name, log fold change, average expression, p-value, Bonferroni-corrected p-value, and genomic coordinates (danRer7/Zv9) as reported by Agilent Feature Extract and annotation files. This file also includes calculated log fold change values for each strain individually by taking the average of the relative log fold change: (AB.TU-AB.WIK)/2 = ABcalc. Calculated strain-specific log fold change values were then centered on zero for each probe (ABcenter). Entrez gene ID, Ensembl ID, gene symbol, and gene name were manually confirmed and harmonized. Several probes annotate to deprecated gene IDs; the few that fall into this category are retained in the file, but identified by strike-through.

SupplementaryDataset5.xls

Differentially expressed mRNA transcripts between AB, TU, and WIK in females. File includes probe name, log fold change, average expression, p-value, Bonferroni-corrected p-value, and genomic coordinates (danRer7/Zv9) as reported by Agilent Feature Extract and annotation files. This file also includes calculated log fold change values for each strain individually by taking the average of the relative log fold change: (AB.TU-AB.WIK)/2 = ABcalc. Calculated strain-specific log fold change values were then centered on zero for each probe (ABcenter). Entrez gene ID, Ensembl ID, gene symbol, and

gene name were manually confirmed and harmonized. Several probes annotate to deprecated gene IDs; the few that fall into this category are retained in the file, but identified by strike-through.

SupplementaryDataset6.xls

Differentially expressed mRNA transcripts between strains regardless of sex. File includes probe name, log fold change, average expression, p-value, Bonferroni-corrected p-value, and genomic coordinates (danRer7/Zv9) as reported by Agilent Feature Extract and annotation files. Log fold change, average expression, p-values, and Bonferroni-corrected p-values are averaged between male and female differential expression datasets (SupplementaryDataset4 and AddtionalFile5).This file also includes calculated log fold change values for each strain individually by taking the average of the relative log fold change: (AB.TU-AB.WIK)/2 = ABcalc. Calculated strain-specific log fold change values were then centered on zero for each probe (ABcenter). Entrez gene ID, Ensembl ID, gene symbol, and gene name were manually confirmed and harmonized. Several probes annotate to deprecated gene IDs; the few that fall into this category are retained in the file, but identified by strike-through.

SupplementaryDataset7.xls

Differentially expressed mRNA transcripts that have corresponding evidence of circadian regulation in 4 mouse liver microarray experiments from the Circadian Expression Profiles Data Base (circaDB, http://circadb.hogeneschlab.org/). File includes a tab for each supplemental dataset (sd1-6) that contains Probeset_ID, Symbol, JTKP, JTKQ, JTKperiod, JTKphase, and Tissue columns. Probeset ID = unique to each microarray expression platform. Symbol = gene symbol. JTKP = JTK_CYCLE p-value. JTKQ = JTK_CYCLE q-value. JTKperiod = period of circadian oscillation, in hours. JTKphase = phase of circadian

oscillation, in hours. Tissue = original dataset where mogene_liver = Mouse 1.OST Liver (Affymetrix), liver = Mouse Liver 48 hour Hughes 2009 (Affymetrix), panda_liver = Mouse Liver Panda 2002 (Affymetrix), and WT_liver = Mouse Wild Type Liver (GNF microarray). “Merge” tab combines all circadian-driven genes from sd1-6 tabs with duplicates removed. “Unique” tab lists the gene symbol for the 82 genes described in this dataset.
